# Supplementary material for: Chromothripsis during telomere crisis is independent of NHEJ, and consistent with a replicative origin
Source: Genome Res. 2019 May;29(5):737–49. doi: 10.1101/gr.240705.118 (PMC6499312; doi:10.1101/gr.240705.118)
Supplement: Supplemental Material [file supp_gr.240705.118_Supplemental_file_1.zip › contigs/annotated_contigs/DB37/contig.2.DB37_length_720_mean_cov_6.72638888889.docx]

**DB37_length_720_mean_cov_6.72638888889**

```
GATCTCTGCACTCTGGGCTTTCTCTAGCTTCCCCAGGGAAGGGAGGCTCGGGGTGAGGTGGGCACGGGGCATCTTTCCTGCCCAACTGT
 >chr17:40672892-40673217 + E=1e-183
GAAGTCCTAAAAAGCTTCACAAAGTTTCTATTGAATGACAGCTTTCTTCTTCTCTTTCTCCAGGGTTGAGTTCCAGAATAAATTCTACA

GCGGGACCGGTTTCAAGTTCTTACCCTTCTCCTTCGAGCATATTCGGGAAGGGAAGTTTGAAGAGTGAGTCCCTGTGAGGGCCGTGTGC

CCCATGCTACCCTCCCCGCCTCCCTCCACAGTGATCAGCTGTGCCTCTCTGCCTGTTG|TA|CTCCTGAGTAGCTGGGACTACAGGCGC
                                                             >chr17:40678890-40679283 +
CCGCCACCATGCCTGGCTAATTTTTTGTATTTTTAGTAGAGACGGGGTTTCACCGTGTTAGCCAGGATGGTCTCAATCTCCTGACCTCG
 E=1e-224
TGATCCGCCCGCCTTGGCCTCCCAAAGTGCTGGGATTGCAGGCATGAGCCACCGCGCCCGGCTGCTCCTGTTTTTAAGAGATGTGCCTT

TACCCTGATCCCCCTCTTGTGGAACCCCAGGACCCCAGACATCTGTCTGATGAGTCTGTGCCCTGGTCCAGGAAATGCCCCTCATCTTG

AGGTCCTAGAACCAGTGTGGGAGAGGGTGGCCTTGGCATGCTTGGCCTGGGGAGTCCCAAGCTTTGTAGAGATAGGGGACAAGTCTATC

ACCACCTTCA
```
